# Dyslipidemia, Diabetes and Atherosclerosis: Role of Inflammation and ROS-Redox-Sensitive Factors

**DOI:** 10.3390/biomedicines9111602

**Published:** 2021-11-03

**Authors:** Elham Hasheminasabgorji, Jay C. Jha

**Affiliations:** 1 Department of Clinical Biochemistry and Medical Genetics, Molecular and Cell Biology Research Center, Faculty of Medicine, Mazandaran University of Medical Sciences, Sari 4815733971, Iran; elham.hasheminasabgorji@gmail.com; 2 Department of Diabetes, Central Clinical School, Monash University, Melbourne 3004, Australia

**Keywords:** diabetes, atherosclerosis, dyslipidemia, inflammation, redox sensitive factors

## Abstract

The prevalence of diabetes is growing at an alarming rate with increased disability, morbidity, and often premature mortality because of the various complications of this disorder. Chronic hyperglycemia, dyslipidemia, and other metabolic alterations lead to the development and progression of macro- and microvascular complications of diabetes including cardiovascular, retinal and kidney disease. Despite advances in glucose and lipid lowering treatments, a large number of diabetic individuals develop one or more types of these complications, ultimately leading to end-organ damage over the time. Atherosclerosis is the major macro-vascular complications of diabetes and the primary underlying cause of cardiovascular disease (CVD) posing heavy burden on the health care system. In this review, we discuss the involvement of dyslipidemia in the progression of atherosclerosis by activating the pro-inflammatory cytokines and oxidative stress-related factors. In addition, we also provide information on various pharmacological agents that provides protection against diabetic atherosclerosis by reducing inflammation and oxidative stress.

## 1. Introduction

**Figure 1:**
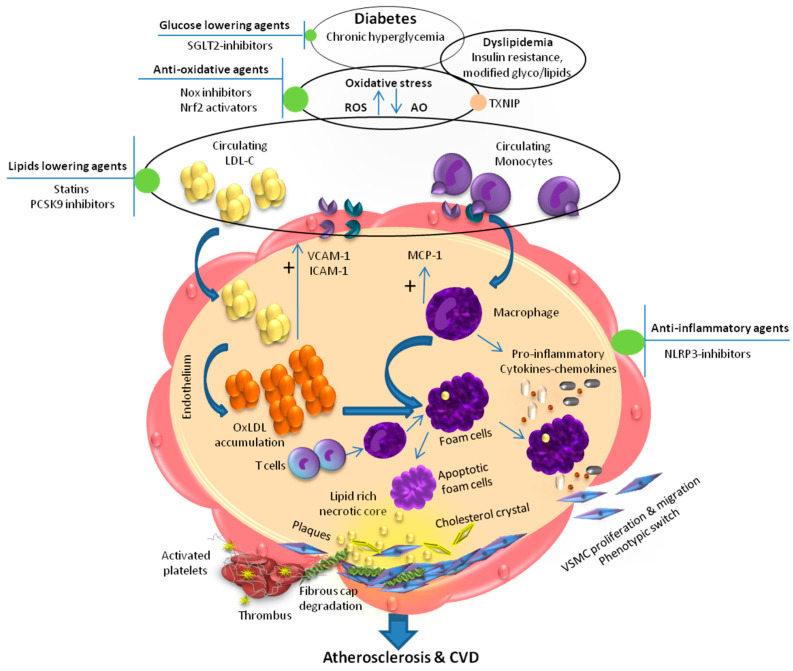
Mechanism of diabetes associated endothelial dysfunction, atherosclerotic cardiovascular disease and anti-atherosclerotic targets: Chronic hyperglycemia and dyslipidemia lead to enhanced oxidative stress resulting in increased ox-LDL formation, activation of immune cells, and upregulation of VCAM-1, ICAM-1, and MCP-1 that promote foam cell formation. Eventually, foam cells undergo apoptosis and form a necrotic core. Accumulation of apoptotic foam cells, and cholesterol crystals lead to formation of atherosclerotic lesion. These pathological events result in proliferation and migration of VSMC and thereby contributing in the formation of lipid rich necrotic core, which in turn promote atherosclerotic lesion development leading to atherosclerotic cardiovascular disease. Upward arrows represent increase and downward arrows represent decrease. SGLT2, Sodium–glucose co-transporter 2; TXNIP, thioredoxin-interacting protein; AO, antioxidant; ROS, reactive oxygen species; NOX, nicotinamide adenine dinucleotide phosphate; Nrf2, NF-E2-related factor-2; PCSK9, proprotein convertase subtilisin/kexin 9; LDL-C, low-density lipoprotein cholesterol; NLRP3, NLR family pyrin domain containing 3; MCP-1, monocyte-chemoattractant protein-1; VCAM-1, vascular cell adhesion molecule-1; ICAM-1, intercellular adhesion molecule 1; oxLDL, oxidized low density lipoprotein; VSMC, vascular smooth muscle cell; CVD, cardiovascular disease.

Diabetes is associated with accelerated atherosclerosis leading to widely distributed vascular lesions including cardiovascular disease (CVD), coronary artery disease, cerebrovascular disease and peripheral arterial disease [1,2,3] with CVD being the major cause of premature death in diabetes [4]. Chronic hyperglycemia, dyslipidemia, and insulin resistance as well as glyco/lipoxidation end-products are the typical uppermost mediators of diabetes causing vascular complications (Figure 1). Dyslipidemia is one of the important risk factors in the development of atherosclerosis, with chronic accumulation of lipid-rich plaque in arteries [5,6]. Both type 1 and type 2 diabetic patients are at high risk of developing lipid metabolism aberrations, however, dyslipidemia is more prevalent among patients with type 2 diabetes [7,8]. Dyslipidemia comprises unhealthy levels of one or more types of lipid in the circulation including high levels of triglycerides and total cholesterol, an increase in numbers of small dense low-density lipoprotein particles, and low levels of high-density lipoprotein cholesterol (HDL-C). Some diabetic individuals may have high levels of low-density lipoprotein cholesterol (LDL-C). However, preponderance of small dense LDL particles appear to be more atherogenic [9]. The exact mechanism of lipoprotein abnormalities in diabetes is poorly understood. Insulin resistance has been implicated in the pathogenesis of diabetic dyslipidemia with increased production of small dense LDL particles and impaired glucose tolerance in type 2 diabetes.

Epidemiological studies have shown correlation between dyslipidemia and the state of metabolic control, age, gender, and body mass index (BMI) in individuals with diabetes [10].

Indeed, numerous studies have shown higher prevalence of dyslipidemia with poor glycemic control, increasing plasma levels of glycated hemoglobin A1c (A1c) and hypercholesterolemia in diabetic individuals [11,12]. Dyslipidemia amplifies the risk of developing atherosclerosis and subsequently CVD in diabetes. Indeed, a significant association was observed between dyslipidemia with high level of total cholesterol/HDL-C ratio, non-HDL-C, and triglycerides and arterial stiffness leading to the development of atherosclerosis in diabetic patients compared to non-diabetic individuals [5,6]. Experimental evidence suggests that in diabetes chronic dyslipidemia, glyco/lipoxidation end-products and reactive oxygen species (ROS) stimulate both immune cells and resident vascular cells to release cytokines through activation pro-inflammatory molecules leading to the development and progression of atherosclerosis [13,14]. One of the earliest events in the pathogenesis of atherosclerosis is lipid accumulation in the arterial wall and formation of foam cells through uptake of modified or oxidized low density lipoprotein (oxLDL) by circulating monocyte and lymphocyte-derived macrophages in the endothelium [15,16].

## 2. Dyslipidemia and Inflammation in Diabetic Atherosclerosis

Dyslipidemia and chronic inflammation are considered as the major driving factors in the plaque formation leading to the sclerosis of blood vessels in diabetes. Atherosclerosis is accompanied by a local inflammation in the vascular wall due to endothelial dysfunction and vascular smooth muscle cells plasticity [17]. Indeed, atherosclerotic plaques formation is a sequential event in which high levels of lipids, particularly LDL cholesterol interacts with both immune and non-immune vascular resident cells including endothelial, smooth muscle cells and macrophages resulting in secretion of growth factors and adhesion molecules leading to formation of foam cells [13,15,16]. It is believed that components of diabetic milieu, oxidative stress and other factors cause damage to vascular endothelial cells leading to the increased expression of adhesion molecules including intercellular adhesion molecule 1 (ICAM-1) and vascular cell adhesion molecule-1 (VCAM-1) and the secretion of chemokines resulting in adhesion of circulating monocytes to the injured area. The attached monocytes penetrate the subendothelial space differentiating into macrophages that release more cytokines including interleukins such as interleukin-1β (IL-1β) and interleukin-18 (IL-18), tumour necrosis factor-α (TNF-α) and interferon-γ (INF-γ) leading to the secretion of chemokines such as monocyte-chemoattractant protein-1 (MCP-1) [18,19]. Subsequently, LDL cholesterol infiltrates the subendothelial space in the intima where it is modified into oxLDL. The oxLDL is then taken up by macrophages leading to the formation of foam cell, lipid-rich necrotic core, atherogenesis and plaque formation [20]. In addition, plasticity of vascular smooth muscle cells including increased transmigration; proliferation and differentiation lead to increase synthesis and deposition of extracellular matrix such collagens, elastin and fibronectin causing intimal thickening, sclerosis and fibrous cap formation [21]. In addition to monocytes, the involvement of circulating T cells is also evident in the development of atherosclerosis in diabetes [14,22]. It is reported that in response to diabetic milieu, T cells regulates the activation of macrophages and the release of cytokines as well as increase expression of chemokines and adhesion molecules which result into infiltration of more immune cells establishing a vascular inflammatory cycle as well as enhancing necrotic core formation. Together the inflammatory environment promotes the development of plaque formation and the progression of atherosclerosis [23]. Subsequently, increase macrophage proteolytic activity leads to plaque destabilization and degradation of the stable fibrous cap resulting in plaque rupture and thrombus formation (Figure 1).

It is also demonstrated that dysregulation of cholesterol efflux signaling in diabetes contribute to the activation of Toll-like receptor-4 (TLR-4), NLR family pyrin domain containing 3 (NLRP3) inflammasome and nuclear transcription factor NF-κB resulting in enhanced inflammation and subsequent development of atherogenesis [17]. In particular, oxLDL and crystalline cholesterol have been linked to activation of NLRP3 inflammasome leading to vascular inflammation [24]. In addition, NLRP3 inflammasomes are reported to enhance macrophages apoptosis via regulation of JNK-induced apoptotic pathway. Furthermore, activation of NLRP3 inflammasome shown to be associated with development and progression of plaque formation and atherosclerosis through increased production of IL-1β and IL-18, upregulation of MCP-1 and VCAM-1 resulting in increased accumulation of vascular extracellular matrix [24]. On the other hand, studies showed negative correlation between HDL and LDL cholesterol efflux could suppress foam cells formation and thereby reducing inflammation and the risk of CVD [17,25,26,27]. In contrary, a recent bulk RNA sequencing data on atherosclerotic lesions in murine model demonstrated that intimal non-foamy macrophages express more inflammatory genes whereas foamy macrophages express more lipid-processing genes [28].

A significant association is apparent between dyslipidemia including elevated level of total cholesterol/HDL-C ratio, non-HDL-C, and triglycerides and arterial stiffness leading to the development of atherosclerosis and higher risk of CVD mortality in diabetic patients compared to non-diabetic individuals [5,6,7]. In addition, ratio of dyslipidemia and hypertension was found to be higher in these diabetic patients. Elevated plasma levels of both small dense LDL (sdLDL) and the LDL cholesterol regulator, proprotein convertase subtilisin/kexin 9 (PCSK9) were observed in diabetic patients with poor glycemic control, insulin resistance and lower levels of HDL, indicating the role for PCSK9 in dyslipidemia and atherosclerosis [29,30]. In newly diagnosed type 2 diabetic patients, a positive association was observed among PCSK9 serum levels with carotid intima-media thickness brachial-ankle pulse wave velocity , C- reactive protein (CRP), neutrophil, lymphocyte, and white blood cell count , thus serving as an early biomarker arteriosclerosis [31]. Therefore, monitoring biochemical parameters such as lipid profiles and glycemic state and their association with dyslipidemia in diabetic patients can reduce prevalence of diabetic cardiovascular diseases [32,33,34].

## 3. Dyslipidemia and ROS-Redox-Sensitive Factors in Diabetic Atherosclerosis

Intra-vascular oxidative stress due to aberrant antioxidant activity and excessive production of reactive oxygen species (ROS) is linked to inflammation and associated atherosclerosis in diabetes [1,35]. In the last decade, significant progress has been made to better understand the underlying mechanism of oxidative stress mediated vascular injury in diabetes. It has been demonstrated that chronic hyperglycemia and dyslipidemia in diabetes increases intravascular ROS production through the activation of various ROS producing enzymes. Indeed, activation of xanthine oxidase (XO), enzymes of the mitochondrial respiratory chain, nicotinamide adenine dinucleotide phosphate (NADPH) oxidases (NOX), cyclooxygenase and uncoupled endothelial nitric oxide synthase (eNOS) as well as modulation of ROS-redox-sensitive factors such as protein kinase C (PKC) and a metabolic gene implicated in redox balance, thioredoxin-interacting protein (TXNIP) have been reported to be relevant in the pathogenesis of atherosclerosis in diabetes [36,37,38].

The ROS-sensitive factors also enhance the production of glyco/lipoxidation end-products including advanced glycation end-product (AGE) and oxidized LDL thereby causing damage to the endothelium, as well as increasing intravascular inflammation and leukocyte recruitment which further contributes to endothelial dysfunction [1,39]. Vascular ROS generation is markedly increased in diabetes via accumulation of AGEs and activating the cellular receptor for AGE (RAGE), which contribute to cytokine secretion and stimulate oxidizing intermediates in hyperglycemic condition [38,40,41]. Studies have shown the link among AGE-RAGE axis and atherosclerosis in diabetes. Indeed, inhibition of RAGE in diabetic ApoE knockout (ApoE KO) mouse showed attenuation of diabetes induced atherogenesis via deactivation of AGE-RAGE signaling and reduction in oxidative stress [42,43].

Increased expression of vascular XO mediated superoxide and hydrogen peroxide production has been demonstrated in human atherosclerotic plaque and endothelial dysfunction [44]. In addition, excess superoxide rapidly inactivates NO by uncoupling of eNOS leading to accelerated endothelial dysfunction, inflammation and atherogenesis [45]. Enhanced level of vascular ROS in diabetic patients with dyslipidemia was found to be associated with increased expression and activity of cyclooxygenase-2 (COX-2) leading to secretion of prostaglandin E_2_ (PGE_2_) and thereby promoting inflammation and atherosclerotic lesion formation [34,46]. Human vascular wall expresses Nox1, Nox2, Nox4, and Nox5 isoform of NADPH oxidase [1]. However, in experimental murine model of diabetes, Nox1 appears to be most deleterious in the pathogenesis of atherosclerosis [47], whereas, Nox4 has been demonstrated to be atheroprotective [48]. Insulin deficient or high fat fed diabetic ApoEKO mice display increased accumulation of cholesterol ester-enriched particles in the blood leading to increased atherosclerotic plaque formation and thereby resulting in accelerated atherosclerosis. In addition, lesion distribution in diabetic ApoEKO mice are similar to humans, with a predominance in the aortic root, carotid artery, and aortic branches [49]. Therefore, ApoEKO mouse represent an ideal model for the study of diabetic atherosclerosis. Experimental evidence also demonstrates that atherosclerosis in diabetic ApoEKO mouse is associated with enhanced vascular inflammation and ROS formation/oxidative stress. Indeed, targeting the source of ROS producing enzyme such as NOX1 in diabetic ApoEKO mouse showed attenuated inflammation and atherosclerosis via reduction in ROS formation, suggesting the role for oxidative stress in atherosclerosis [47]. Global genetic deletion of Nox1 in ApoE KO deficient diabetic mice showed decreased macrophage infiltration and reduced plaque lesion size at the aortic valve through reduction in ROS formation, suggesting the pathological relevance of Nox1 in atherosclerosis [47,50]. Partial inhibition of Nox2 by gp91dstat not only reduced superoxide production but also reduced lipid deposition and atherosclerosis. Conversely, complete deficiency of Nox2 elevated lipid deposition and vascular dysfunction, while decreased superoxide generation [51], with increased mortality [47] suggesting that Nox2 shouldn’t be targeted. On the other hand, Nox4 demonstrated vascular protection [52] via NF-E2-related factor-2 (Nrf2) activation and negative regulation of NF-κB [53]. Nox5 is present in humans but not in rodents [54,55] and therefore it has been difficult to study the role of Nox5 in conventional rodent models of diabetes. Nevertheless, increased mRNA and protein expression of Nox5 has been reported in patients with coronary artery disease and atherosclerotic lesions compared with control group [56]. In pathological condition, lysophosphatidylcholine (LPC) induces calcium influx and subsequently activation of NOX5 which promotes endothelial oxidative stress and atherosclerotic lesions [54]. In addition, intracellular Ca^2+^ stimulates the activity of Nox5, thus leading to increased ROS production resulting in cardiac hypertrophy in cardiomyocyte-specific transgenic Nox5 mouse model [57].

The activation of PKC signaling is linked to NADPH oxidase derived ROS/oxidative stress, inflammation and atherosclerosis in diabetes [38,40,43,46]. Indeed, it has been reported that PKC-β promotes endothelial dysfunction leading to increased VCAM-1 expression, and monocyte-macrophage derived foam cell formation resulting in atherosclerotic plaque formation in ApoE KO diabetic mouse [58,59]. In addition, inhibition of NADPH oxidase activity by Apocynin treatment showed diminished vascular superoxide generation and suppression of PKC activity in animal model of diabetes [36,46]. Another ROS-redox-sensitive factor TXNIP is found to be associated with enhanced oxidative stress and inflammation leading to atherosclerosis in ApoE KO mouse model of diabetes. Indeed, deficiency of TXNIP led to attenuation of atherosclerosis via reduction in oxidative stress in experimental diabetes [60]. Imbalances in antioxidants and ROS production leads to oxidative stress and has been linked to the development of atherosclerosis in diabetes. Indeed, deficiency of glutathione peroxidase (GPx) appears to enhance atherogenesis in diabetes. Gpx1 deficient apo E^−/−^ diabetic mice showed increased NADPH activity as well as accelerated atherosclerotic plaque size [42]. In addition, Gpx1 deficient diabetic mice showed increased oxidation of LDL and accelerated the development of atherosclerosis [61]. Similarly, a decreased level of plasma GPx3 was found to be associated with increased carotid atherosclerosis in type 2 diabetes patients [51]. Prevalence of oxidative damage has been shown to be increased in type 2 diabetic patients with high ischemia modified albumin (IMA) levels and low HDL-C concentrations. HDL-C found to be inversely correlated with the occurrence of oxidative damage in type 2 diabetic patients [62]. However, prolonged inflammation, glycation, and oxidative modifications could result in the loss of antiatherogenic capacity of HDL-C in diabetes, leading to dysregulation of cholesterol efflux, impairment of HDL antioxidative properties, over production of ROS, and development of atherosclerosis [63].

## 4. Therapeutic Approaches to Combat Atherosclerosis in Diabetes

Despite extensive advancement in therapeutic strategies to prevent and treat vascular complication of diabetes, CVD is the leading cause of morbidity and mortality globally [1,3]. As mentioned earlier, dyslipidemia, inflammation, oxidative stress, and glycemic index are associated with endothelial dysfunction leading to progression of vascular complication of diabetes. Therefore, therapeutic approaches targeting these pathophysiological mechanisms could be promising strategies to combat the diabetes associated vascular complications including CVD and atherosclerosis [64,65].

### 4.1. Glucose-Lowering Agents

Epidemiological studies have demonstrated a strong association between various indices of glycemia (fasting plasma glucose, post-prandial or post oral glucose challenge plasma glucose, HbA1c) and risk of atherosclerotic cardiovascular disease in diabetes. The benefits of glucose-lowering agents in combination with lipid-lowering agents and antihypertensive therapy are more prevalent in reducing diabetic microvascular complications compared to macrovascular complications. However, recent studies, particularly the use of sodium–glucose co-transporter 2 (SGLT2) inhibitors have shown promising outcomes in reducing macrovascular complications in patients with type 2 diabetes [66]. SGLT2 inhibitors function through a novel mechanism of reducing renal tubular glucose reabsorption leading to a reduction in blood glucose without stimulating insulin release. SGLT2 inhibitors including empagliflozin, canagliflozin, and dapagliflozin lower blood glucose via increasing glucose excretion in urine in type 2 diabetic patients [66,67,68]. It has been revealed that SGLT2 inhibitors could have anti-inflammatory and antioxidative properties. Empagliflozin was reported to attenuate adverse cardiovascular events and was associated with lower blood pressure and arterial stiffness in type 2 diabetic patients [67]. Furthermore, canagliflozin significantly reduced the rate of cardiovascular mortality and morbidity in type 2 diabetic patients. Similarly, dapagliflozin administration resulted in reduced atherosclerotic plaque formation, ROS production, and inactivation of NLRP3 inflammasome with decreased secretion of cytokines IL-1β and caspase-1 [1,3,46,50].

### 4.2. Lipid-Lowering Agents

The management of diabetic dyslipidemia to achieve improvements in lipid profiles usually requires a combination of non-pharmacological including medical nutrition therapy, weight loss, and physical activity along with pharmacological therapy. However, the current management of diabetic dyslipidemia generally is not optimal. Aggressive therapy of dyslipidemia, particularly the cholesterol-lowering agents have been shown to be effective in reducing the risk and progression of CVD in patients with diabetes [69]. The most commonly used lipid-lowering pharmacological agents include statins, fibrates, cholesterol absorption inhibitors, omega-3 fatty acids and PCSK9 inhibitors.

#### 4.2.1. Statins

Inhibition of 3-hydroxymethylglutaryl coenzyme A (HMG-CoA) reductase by statin reduces the cholesterol synthesis in the liver leading to upregulation of LDL receptors which results in lower level of plasma LDL [70]. Cholesterol-lowering agent, statin has been shown to be associated with reduced cardiovascular risk along with significant reduction in atherosclerosis in patients with diabetes [65,71]. Statin mediated lower level of plasma LDL cholesterol is also linked to decreased triglycerides and increased HDL-cholesterol as well as improved endothelial function and decreased vascular inflammation and oxidative stress [72,73]. The recommendation from American Diabetes Association guidelines suggest that in addition to lifestyle modification, diabetic patients with atherosclerotic cardiovascular disease should be treated with high-intensity statins to achieve 50% reduction in LDL-cholesterol [69]. Indeed, several clinical trials in diabetic patients showed statins therapies significantly reduced cardiovascular risk and CVD mortality as well as ischemic stroke [74,75]. A randomized clinical trial study showed that high-intensity statin therapy using rosuvastatin 40 mg or atorvastatin 80 mg for 24 months significantly altered the progressive nature of diabetic coronary atherosclerosis, yielding regression of disease in diabetic and non-diabetic patients [76]. In contrary, higher dose of statins can cause some side effects including myalgia, myositis, rhabdomyolysis, and elevation in liver enzymes [77,78] as well as new onset diabetes, particularly in individuals with metabolic syndrome (BMI > 30 and A1c > 6%) [79].

#### 4.2.2. Fibrates

Activation of nuclear peroxisome proliferator-activated receptor alpha by fibrates can reduce triglyceride level via stimulation of lipoprotein lipase activity. The use of fibrates have been demonstrated to be associated with upregulation of lipoproteins apoA-1 and A-II along with lower level of small dense LDL and higher level of HDL [80,81]. The Diabetes Atherosclerosis Intervention Study (DAIS) in 418 diabetic patients showed that treatment of fenofibrate group significantly reduced the rate of progression of coronary atherosclerosis [82]. It was also suggested that fibrates either as monotherapy or in combination with statins can be effective in the treatment of diabetic dyslipidemia. However, The Fenofibrate Intervention and Even Lowering in Diabetes (FIELD), a randomized controlled trial study in 9795 patients with type 2 diabetes found no significant reduction in reducing macro and microvascular complications after 5-year follow-up treatment with fenofibrate [83].

#### 4.2.3. PCSK9 Inhibitors

Another lipid-lowering agents, PCSK9 inhibitors including Alirocumab and Evolocumab are shown to be effective in lowering LDL-C significantly when used as monotherapy or in combination with statins. PCSK9 inhibitors prevent binding of PCSK9 with LDL receptors leading to upregulation of LDL receptors with resultant decrease in circulating LDL-cholesterol level [84]. As per the Institute for Health and Care Excellence (NICE) guidelines, PCSK9 inhibitors alirocumab and evolocumab are recommended for patients with primary hypercholesterolemia or mixed dyslipidemia that is not controlled with statins. PCSK9 inhibitors are recommended in patients with atherosclerotic CVD who have LDL-C ≥ 70 mg/dL or non-HDC ≥ 100 mg/dL and are on maximum tolerated dose of statin therapy [84].

Indeed, PCSK9 inhibitors were found to be associated with atheroma regression and decreased atheroma volume. Glagov randomized clinical trial in 968 patients with CVD, showed subcutaneous injection of evolocumab 420 mg monthly led to lower level of LDL-C and over all decreased total atheroma volume by 5.8 mm [85]. Furthermore, it appears that inhibition of PCSK9 prevent vascular complication of diabetes through LOX1 inhibition [46]. The oxLDL promote vascular inflammation via lectin-like oxLDL receptor 1 (LOX1) activation leading to diabetic atherosclerosis. It was observed that, LDL-C levels reduced by 49.1% in type 2 diabetic patients treated for 24 weeks with alirocumab, a PCSK9 inhibitor. In addition, another PCSK9 inhibitor evolocumab significantly reduces the risk of CVD in both non-diabetic and diabetic patients [50]. The FOURIER trial in 27,564 patients with atherosclerotic CVD and LDL level ≥ 70 mg/dL while being on highly tolerated statin showed benefits of evolocumab subcutaneous injection (140 mg every 2 weeks or 420 mg every month) in reducing LDL-C by 59% in the treatment group compared to placebo. The study reported 15% relative risk reduction in the primary endpoint (composite of cardiovascular death, stroke, myocardial infarction, coronary revascularization, and hospitalization from unstable angina). Moreover, in a subsequent study, administration of evolocumab in 11,031 diabetic patients showed a significant risk reduction in the above composite primary endpoint with no increase in new onset diabetes or any deleterious effect on glycaemia [86]. Despite the strong benefits in lowering LDL-C and thus reducing the CVD, PCSK9 inhibitors are very expensive with the annual cost of >$14,500 and can impose a significant economic burden even in developed countries [87].

### 4.3. Anti-Inflammatory Agents

Activation of NLRP3 inflammasome by exogenous and endogenous danger signals including hyperglycemia, hyperlipidemia, hyperuricemia, lipopolysaccharide (LPS), AGEs and mitochondrial ROS stimulates the inflammatory cascade reaction in diabetes. Activation of NLRP3 inflammasome with enhanced release of inflammatory cytokines IL-1β and IL-18 has been implicated in vascular complications of type 2 diabetes disease [43]. MCC950 is one of the most potent and highly specific small-molecule inhibitors of NLRP3 inflammasome. MCC950 prevents the NLRP3 conformational change and subsequent inflammasome formation by abrogating oligomerization and thereby deactivating caspase-1 which blocks the conversion of pro- IL-1β and IL-18 to mature IL-1β and IL-18 leading to reduced inflammation. In a recent study, administration of MCC950 showed downregulation of IL-1β and caspase-1 and improved vascular function and reduced atherosclerosis in ApoEKO diabetic mice [88].

Another anti-inflammatory agent lipoxin has been demonstrated to suppress NF-κB, IL-1β, TNF-a, and TGF-β and thereby attenuating the development of diabetes associated macrovascular complications [1]. Thiazolidinediones including pioglitazone and rosiglitazone were associated with activation of proliferator-activated receptor gamma (PPARγ), leading to negative regulation of NF-κB targets, thus provided protection against oxidative stress, blood pressure, and improved insulin sensitivity compared with control group in type 2 diabetic patients. Also, thiazolidinediones administration lowered the serum CRP and improved endothelial dysfunction [3,89]. In addition, Canakinumab reported to lower fibrinogen, CRP, and IL-6 suggesting a potential role for decreasing cardiovascular risk in diabetic patients [13,89].

### 4.4. Antioxidants and Nrf2 Activators

Several studies revealed that Nrf2 activators such as epigallocatechin3-gallate (EGCG), curcumin, sulforaphane, and 1,2-mercapto-3-sulfur ketone derivatives (oltipraz) could have potential effects on attenuating oxidative stress and reducing endothelial dysfunction in diabetes [37]. Curcumin, a natural compound has been shown to reduce inflammation and oxidative stress. Atherosclerotic lesions and free fatty acids were reduced in animal models treated with curcumin in comparison to untreated groups. Curcumin decreased expression of MCP-1, IL-1β, and TNF-α leading to anti-inflammatory responses in reducing risk of CVD in type 2 diabetic patients [37,55]. Similarly, sulforaphane was shown to suppress ROS production as well as inflammatory factors ICAM-1 and VCAM-1 in experimental diabetic rats [90]. In addition, Oltipraz was able to reduce eNOS generation and insulin resistance in a high-fat diet fed animal model [91]. Administration of another Nrf2 activator EGCG in ApoEKO mouse model fed with a high-fat diet significantly decreased atherosclerotic lesion formation and downregulation of inflammatory cytokines IL-6 and TNF-α as well as reduced level of total and LDL cholesterol while enhancing liver X receptor α (LXRα) and LXRβ and HDL concentrations. These results indicated the anti-atherosclerotic effects of EGCG treatment in diabetes [92].

### 4.5. Agent Targeting the Source of ROS (NOX Inhibitors)

It is believed that targeting the source of ROS could be the ideal approach to alleviate the burden of oxidative stress mediated cellular injury in chronic diseases including diabetic atherosclerosis. The pro-oxidant enzymes NADPH oxidases appear to be the key source of ROS in the vasculature. Several small molecules have been and are still being used as direct NADPH oxidase inhibitors including GKT136901 and GKT137831 developed by GenKyoTex [1,50,93]. Both of these GKT compound specifically inhibit NOX1 and NOX4 and a lesser degree to NOX5. Administration of GKT136901 in ApoEKO mice attenuated ROS generation and atherosclerosis and decreased CD44 expression in atherosclerotic lesions [93]. Another study using GKT137831, a dual inhibitor of NOX1/4 in streptozotocin induced diabetic ApoEKO mouse model showed protection against atherosclerosis by reducing aortic plaque formation and inflammation via reduction in ROS formation [46]. Furthermore, other relatively less specific NOX inhibitors including gp91ds-tat, apocynin, and diphenyleneiodonium were demonstrated to suppress ROS generation, vascular dysfunction and improve blood pressure [56]. In addition, a peptide NOXA1ds designed to inhibit NOX1 found to be associated with reduction in vascular ROS formation albeit in hypoxia-induced or angiotensin II–induced vascular pathology [94]. More research is needed for the validation of NOXA1ds in diabetes-related atherosclerosis and cardiovascular disease.

## 5. Conclusions

It is apparent from both preclinical and clinical studies that diabetes is one of the leading risk factors for the development and progression of atherosclerosis, the underlying cause of increased cardiovascular risk and mortality. The evidence supports how elements of diabetic milieu including dyslipidemia and glycol/lipoxidation end-products amplify the vascular ROS formation, activation of immune cells and inflammation resulting in atherogenesis and plaque formation. Current therapies targeting aforementioned mechanisms including glycemic control, lipid-lowering agents, anti-oxidative, and anti-inflammatory agents show certain level of benefits in reducing the progression of diabetes-related atherosclerosis. However, more studies are needed to identify the novel mechanism based specific targets for the treatment and prevention of diabetes associated atherosclerotic cardiovascular disease.

## Data Availability

Not applicable.
